# Association of NOS3 (rs1799983) and DDAH2 (rs805305) Gene Polymorphisms With Coronary Artery Disease in the Northern Indian Cohort

**DOI:** 10.7759/cureus.79546

**Published:** 2025-02-24

**Authors:** S. M Shiraz Rizvi, Farzana Mahdi, Jyoti Dwivedi, Bashir Ahmad Mir, Zeashan H Zaidi, Namakkal Soorappan Rajasekaran

**Affiliations:** 1 Biochemistry, Era's Lucknow Medical College and Hospital, Era University, Lucknow, IND; 2 Cardiology, Era's Lucknow Medical College and Hospital, Era University, Lucknow, IND; 3 Community Medicine, Era’s Lucknow Medical College and Hospital, Era University, Lucknow, IND; 4 Pathology, University of Alabama at Birmingham, Birmingham, USA

**Keywords:** coronary artery disease, ddah2, endothelial dysfunction, nos3, polymorphism

## Abstract

Introduction and objectives: Nitric oxide synthase (*NOS3*) and dimethylarginine dimethylaminohydrolase 2 (*DDAH2*) polymorphisms are associated with reduced nitric oxide (NO) synthesis and endothelial dysfunction, increasing the risk of cardiovascular disease (CVD). This study aimed to analyze the single nucleotide polymorphism (SNP) of the *NOS3* and* DDAH2* genes and to identify their association with the risk of coronary artery disease (CAD).

Materials and methods: *NOS3* (rs1799983) and *DDAH2* (rs805305) single nucleotide polymorphisms (SNPs) were analyzed in 148 ST-elevation myocardial infarction (STEMI) patients and 75 healthy subjects (control) using polymerase chain reaction-restriction fragment length polymorphism (PCR-RFLP). Results were analyzed using descriptive statistics and summarized as mean ± standard deviation. Chi-square was used to determine the association between variables of interest.

Results: The G894T *NOS3* SNP was significantly linked to STEMI risk (p=0.003), with the TT genotype (20.3%) and T allele (39.5%) more frequent in cases than controls. The TT genotype was strongly associated with increased STEMI risk (OR=4.33 (95% CI: 1.57-12.04), p < 0.0001). For the *DDAH2* SNP, the GG genotype was more common in cases (30.4%) than in controls (20.0%), while the CC genotype was less frequent in cases (16.9%) compared to controls (28.0%) (p=0.026, OR=0.40 (95% CI: 0.17-0.90)).

Conclusion: These findings link the G894T *NOS3* polymorphism to heightened STEMI risk, particularly in patients with diabetes, and highlight the association of *DDAH2* SNPs with CAD, emphasizing the prevalence of GG genotypes in STEMI cases.

## Introduction

Coronary artery disease (CAD) arises from complex genetic and environmental interactions. Among acute coronary syndromes (ACS), ST-elevation myocardial infarction (STEMI) is the most severe, caused by prolonged coronary artery blockage and extensive cardiac damage [1]. Nitric oxide (NO), a crucial signaling molecule, maintains endothelial cell function and plays a key role in CAD pathophysiology. One of the most dominant endogenous vasodilators is NO. It causes inhibition of platelet adhesion and aggregation, inhibits migration of vascular smooth muscle cells, regulates the interaction of vessel and platelet, and decreases the formation of atherogenic oxidized low-density lipoprotein (OxLDL); therefore, dysregulated NO production is associated with endothelial dysfunction and heightened cardiovascular risk [2].

Genetic variations, particularly single nucleotide polymorphisms (SNPs) in genes nitric oxide synthase (*NOS3)* and dimethylarginine dimethylaminohydrolase 2​​​​​​​ (*DDAH2)*, influence CAD susceptibility [3]. The *NOS3* gene, which codes for endothelial nitric oxide synthase (eNOS), is expressed in the endothelium and is located on chromosome number 7q35-36, and it contains 25 introns and 26 exons, which encode for an mRNA of 4052 nucleotides, which leads to the production of a protein eNOS containing 1203 amino acids, which catalyzes the production of NO and L-citrulline from L-arginine [4]. The G894T polymorphism of *NOS3* exon 7 is considered to lead to decreased production of NO, as the variation in genotype changes the linear structure of the protein molecule, and it is likely that it changes some properties of the eNOS enzyme, which leads to its altered function, which leads to damage of the endothelium, and further increases STEMI risk. It has been demonstrated in studies that the eNOS protein, which contains Asp at position 298, undergoes specific proteolysis in the endothelium and vascular tissues [5,6]. Therefore, the cleaved fragments would be likely to have significantly decreased nitric oxide synthase activity. The Glu298Asp SNP also affects the localization of eNOS to the caveolar membrane.

Currently, asymmetrical dimethyl arginine (ADMA) is considered a risk factor for cardiovascular diseases, and subsequent to its entry from blood vessels into the cell, it is metabolized largely by the enzyme dimethylarginine dimethylaminohydrolase (DDAH). ADMA is metabolized to citrulline and dimethylamine by the activity of DDAH [7,8]. The gene encoding DDAH-2 is present on chromosome 6p21.3. Variants like −449 G/C in the promoter region of the *DDAH2* gene are linked to the inheritable risk of CAD and increased prevalence of STEMI [9]. Dysfunctional NO metabolism, a hallmark of CAD, impairs vasodilation and contributes to thrombosis, inflammation, and vascular proliferation, exacerbating STEMI progression [10,11]. *NOS3* gene polymorphisms, such as allelic variants affecting NO production, further illustrate the genetic basis of CAD [12,13].

Understanding genetic determinants, including *NOS3* and *DDAH2* polymorphisms, is critical for identifying at-risk individuals and elucidating mechanisms underlying CAD progression [14]. This study investigates the role of these SNPs in STEMI patients, with and without diabetes, to enhance insights into genetic influences on CAD.

## Materials and methods

Study population

This study assessed the *NOS3* (rs1799983) and *DDAH2* (rs805305) gene SNPs in CAD patients (n=148) and healthy control subjects (n=75) at Era University Hospital, and the study was performed during the period from June 1, 2021 to May 30, 2024. Patients were categorized into STEMI and STEMI with known diabetes mellitus groups. Electrocardiography (ECG) and 2D-echocardiography (2D-ECHO) were done as investigative procedures for the diagnosis of STEMI, and genotyping was performed for all the subjects. Informed consent was obtained from all participants, and the study was approved by the Institutional Ethical Committee of Era University, Lucknow, following ethical guidelines for human subject research.

Selection of STEMI subjects

ST-segment elevation (STE) is a key indicator of complete coronary artery occlusion without collateral circulation, which can lead to irreversible infarction in a significant area of ischemic myocardium, requiring immediate reperfusion therapy [15]. Patients with acute chest pain were evaluated for STEMI using ECG criteria from the American Heart Association, American College of Cardiology, European Society of Cardiology, and World Heart Federation. STE is considered significant when the J point in at least two adjoining leads measures ≥2 mm (0.2 mV) in men or ≥1.5 mm (0.15 mV) in women in leads V2-V3, and ≥1 mm (0.1 mV) in other contiguous leads. STE specificity in STEMI is increased by reciprocal changes (ST depression in leads opposite the major vessel of injury). STEMI is also considered equivalent to a new left bundle branch block. In leads V2-V3, the cutoff for STE is >0.2 mV in men over 40 years, >0.25 mV in men under 40 years, and >0.15 mV in women. The QRS complex aligns with ST-segment elevation of 1.0 mm or more, and Sgarbossa’s criteria are used to assess patients with pre-existing left bundle branch block [16].

Inclusion and exclusion criteria for the study

The study included patients aged 25 to 65 years with clinically relevant STEMI, defined as ≥1 coronary segment with ≥50% stenosis in at least one of the 15 coronary segments. The patients were divided into two groups: those with and without the secondary complication of diabetes mellitus. Control subjects were normal, healthy adults, and the patients were willing and able to provide written consent; they were also matched with case groups for age and gender. Patients with stenosis <50%, critical illness, septicemia, malignancy, pregnancy, acute or chronic kidney disease, cerebrovascular accidents, immunocompromised conditions, and those under 25 years of age were excluded from the study.

Data collection and blood sampling

Clinical, biochemical, and molecular investigations were conducted to gather data on patient demographics, history of secondary complications (such as diabetes), family history, SNPs, and associated clinical factors. After obtaining informed consent, a 2 mL blood sample was collected from each patient into the vial containing ethylenediaminetetraacetic acid (EDTA). The EDTA vial was stored at −20°C for genotype analysis.

Polymerase chain reaction-restriction fragment length polymorphism (PCR-RFLP)

NOS3 Genotype Identification at the rs1799983 Locus

**Figure 1:**
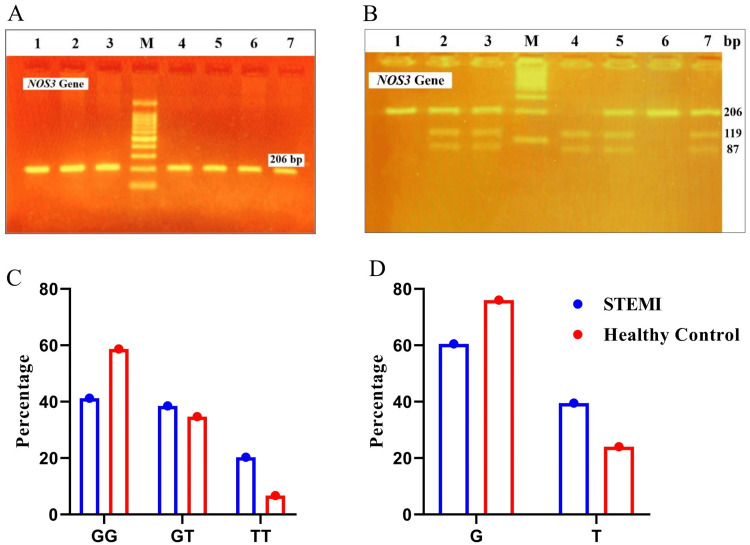
Intergenotypic comparison of the NOS3 gene in STEMI patients and healthy controls A: PCR product image of various samples analyzed using 2% agarose gel electrophoresis, showing a 206 bp amplicon of the *NOS3* gene spanning polymorphic site (rs1799983) (Lanes with amplicons are labeled from 1 to 7 and Lane M represents – 100 bp DNA size marker). B: Restriction fragment length polymorphism (RFLP) analysis of the G/T polymorphism in exon 7 of the *NOS3* gene, using 3% agarose gel electrophoresis after MboI digestion of the PCR product. Lane M: DNA size marker - 100 bp; Lane 1 and 6: GG (206 bp); Lane 2, 3, 5, and 7: GT (206 bp, 119 bp, and 87 bp); Lane 4: TT (119 bp and 87 bp). C: A bar graph comparing the percentage of genotypes of the *NOS3* gene between STEMI and healthy control groups, where the GG genotype was less frequent in STEMI cases (41.2%) than in controls (58.7%), while the GT genotype in STEMI (38.5%) showed no significant difference with controls (34.7%). The TT genotype was significantly more common in STEMI (20.3%) compared to controls (6.7%). D: A percentage comparison of allele frequencies for the G/T polymorphism of the *NOS3* gene between the STEMI and healthy control groups, where the T allele was more frequent in STEMI cases (39.5%) than in controls (24.0%), while the G allele was less common in cases (60.5%) than in controls (76%). STEMI: ST-elevation myocardial infarction; NOS3: nitric oxide synthase; PCR: polymerase chain reaction

Polymerase chain reaction (PCR) was used to amplify the polymorphic region of the *NOS3* gene at the +894 (G/T) position (Figure 1A), followed by restriction fragment length polymorphism (RFLP) analysis. The PCR reaction was performed in a 20 µL volume, containing 2 µL of genomic DNA, 10 µL of Taq PCR mix (Takara) with 1 mmol/L MgCl_2_, 100 mmol/L dNTPs, and 0.5 U Taq polymerase, and 2 µL of the following primers (IDT): Forward: 5’-CAT GAG GCT CAG CCC CAG AAC-3’ and Reverse: 5’-AGT CAA TCC CTT TGG TGC TCA C-3’ and 6 µL double distilled water. Amplification was performed of extracted DNA with a concentration of 50-100 ng/µL, and the PCR conditions used were denaturation at 95ºC for five minutes, followed by 40 cycles of 94ºC for 30 seconds, 66ºC for 30 seconds, 72ºC for 30 seconds, and final extension at 72ºC for eight minutes. Digestion was performed with the MboI (New England Biolabs, MA, USA) restriction enzyme. This generated three profiles: homozygous wild-type GG (one 206 bp fragment), heterozygous GT (three fragments: 206, 119, and 87 bp), and homozygous mutant TT (two fragments: 119 and 87 bp) (Figure 1B) [10]. The digested products were separated by 3% agarose gel electrophoresis, stained with ethidium bromide (EtBr), and visualized under UV light.

DDAH2 Genotype Identification at the rs805305 Locus

**Figure 2:**
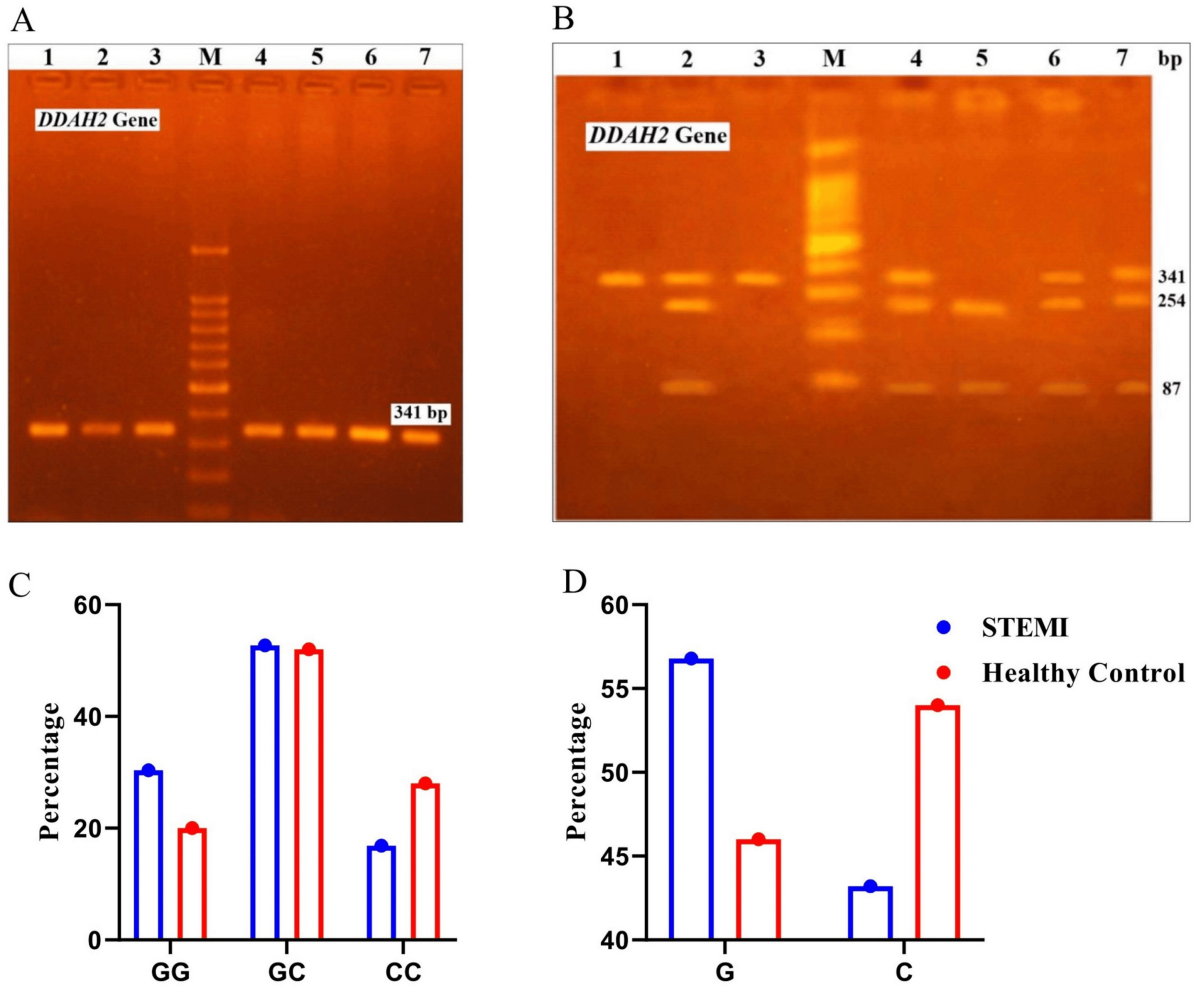
Genotype and allele distribution of the DDAH2 gene in STEMI patients and healthy controls A: PCR product image of various samples analyzed using 2% agarose gel electrophoresis, showing a 341 bp amplicon of the *DDAH2* gene spanning polymorphic site (rs805305) (Lanes with amplicons are labeled from 1 to 7, and Lane M represents – 100 bp DNA size marker). B: Restriction fragment length polymorphism (RFLP) analysis of the G/C polymorphism at the rs805305 locus in the *DDAH2* gene, using 3% agarose gel electrophoresis after SmaI digestion of the PCR product. M: DNA size marker - 100 bp; Lane 1 and 3: GG (341 bp); Lane 2, 4, 6, and 7: GC (341 bp, 254 bp, and 87 bp); Lane 5: CC (254 bp and 87 bp). C: A bar graph comparing the percentage of genotypes of the *DDAH2* gene between STEMI and healthy control groups, where the GG genotype was more frequent in STEMI (30.4%) than in controls (20.0%) while the GC genotype in STEMI (52.7%) showed no significant difference with controls (52.0%). The CC genotype was less common in STEMI (16.9%) compared to controls (28.0%). D: A percentage comparison of allele frequencies for the G/C polymorphism of the *DDAH2* gene between the STEMI and healthy control groups, where the G allele was more frequent in STEMI cases (56.8%) than in controls (46.0%), while the C allele was less common in cases (43.2%) than in controls (54%). STEMI: ST-elevation myocardial infarction; DDAH2: dimethylarginine dimethylaminohydrolase 2; PCR: polymerase chain reaction

The PCR-RFLP method was used to genotype ​​*DDAH2* at the rs805305 locus. The reaction mix included 2 μL genomic DNA, 10 µL of Taq PCR mix (Takara) with 1 mmol/L MgCl_2_, 100 mmol/L dNTPs and 0.5 U Taq polymerase, and 2 µL of the primers (IDT) and 6 µL double distilled water to a final volume of 20 μL. The target region was amplified with primers (Forward: 5’-CCT TCT CGT TCG GGT ATT CAG-3’ and Reverse: 5’-TCC AGA CCT TCC GCT CCT-3’). PCR conditions: denaturation at 95ºC for one minute, followed by 45 cycles of 95ºC for 20 seconds, 64ºC for 20 seconds, 72ºC for 30 seconds, and final extension at 72ºC for six minutes (Figure 2A). The 341 bp PCR products were digested with 5 U of SmaI (New England Biolabs) restriction enzyme and incubated overnight at 37ºC. Digested products were separated by 3% agarose gel electrophoresis. The resulting bands were wild-type GG (341 bp), heterozygous GC (341, 254, and 87 bp), and homozygous CC (254 and 87 bp) (Figure 2B) [17].

Statistical analysis

Statistical analysis was performed using IBM SPSS Statistics for Windows, Version 21 (Released 2012; IBM Corp., Armonk, New York, United States). The chi-square test (χ²) with a p-value < 0.05 was used to assess the association between genotypes and risk factors. Hardy-Weinberg Equilibrium (HWE) was tested for both case groups (Group I: STEMI and Group II: STEMI with diabetes mellitus) and controls (Group III: healthy subjects). Odds ratios (OR) with a 95% confidence interval (CI) were calculated to evaluate the association between genotypes and CAD risk, with significance set at p < 0.05.

## Results

The study compares demographic variables between ST-elevation myocardial infarction (STEMI) cases and healthy controls (Table 1). The mean age of STEMI patients was 53.7 ± 8.0 years, compared to 52.6 ± 8.0 years in controls, with no significant difference (t=1.01, p=0.315). Among STEMI cases, 62.2% (n=92) were male and 37.8% (n=56) female, while controls comprised 64% (n=48) males and 36% (n=27) females, showing no significant difference in sex distribution (chi-square=0.07, p=0.789). These results suggest that the groups are well-matched in terms of age and sex, making them suitable for comparison.

**Table 1:** Comparison of demographic variables between cases of STEMI (n=148) and healthy controls (n=75) STEMI: ST-elevation myocardial infarction

Variable		Case (STEMI)	Control (Healthy)	Sig.
Age	Mean±SD years	53.7±8.0	52.6±8.0	t=1.01, p=0.315
Sex	Male	92 (62.2%)	48 (64%)	chi sq=0.07, p=0.789
	Female	56 (37.8%)	27 (36%)	

Next, we examined the association of *NOS3* and *DDAH2* gene variants with STEMI risk compared to healthy controls (Table 2). For the *NOS3* SNP, the GG genotype was less frequent in STEMI cases (41.2%) (n=61) than in controls (58.7%) (n=44), while the GT genotype showed no significant difference (p=0.136). The TT genotype, however, was significantly more common in STEMI patients (20.3%) (n=30) compared to controls (6.7%) (n=5) (p=0.003, OR=4.33) (Figure 1C). Similarly, the T allele was more frequent in STEMI cases (39.5%) (n=117) than in controls (24.0%) (n=36) (p=0.001, OR=2.07) (Figure 1D). For the *DDAH2* SNP, the GG genotype was more frequent in STEMI (30.4%) (n=45) than in controls (20.0%) (n=15), while the CC genotype was less common in STEMI (16.9%) (n=25) compared to controls (28.0%) (n=21) (p=0.026, OR=0.40), suggesting the CC genotype is non-pathogenic (Figure 2C). Additionally, the G allele was more prevalent in STEMI cases (56.8%) (n=168) than the C allele (43.2%) (n=128) (p=0.032, OR=0.65) (Figure 2D) (Table 3). These results highlight significant genetic differences associated with STEMI risk.

**Table 2:** Shows 95% CI comparison of genotypic variants between STEMI and healthy controls in relation to NOS3 and DDAH2 single nucleotide polymorphism The statistical test used was chi-square (X^2^) to obtain the p-values. Ref. shows the reference category, comparative to this category all the p-values and risk ratios were calculated. STEMI: ST-elevation myocardial infarction; SNP: single nucleotide polymorphism; *NOS3*: nitric oxide synthase; *DDAH2*: dimethylarginine dimethylaminohydrolase 2

Gene		Case (STEMI) (n=148)		Control (Healthy) (n=75)		Significance		OR (95% CI)
		No.	%	No.	%	chi sq	p-value	
*NOS3* SNP (rs1799983)	Genotype							
	Wild GG	61	41.2%	44	58.7%	Ref.	‘--	1.00
	Heterozygous mutant GT	57	38.5%	26	34.7%	2.22	0.136	1.58 (0.86-2.89)
	Homozygous mutant TT	30	20.3%	5	6.7%	8.80	0.003	4.33 (1.57-12.04)
*DDAH2* SNP (rs805305)	Genotype							
	Wild GG	45	30.4%	15	20.0%	Ref.	‘--	1.00
	Heterozygous mutant GC	78	52.7%	39	52.0%	1.30	0.254	0.67 (0.33-1.34)
	Homozygous mutant CC	25	16.9%	21	28.0%	4.95	0.026	0.40 (0.17-0.90)

**Table 3:** Shows 95% CI comparison between STEMI and healthy controls in relation to NOS3 and DDAH2 allele frequency Statistical analysis was done using the chi-square (X^2^) test to obtain the p-values. SNP: single nucleotide polymorphism; STEMI: ST-elevation myocardial infarction; *NOS3*: nitric oxide synthase; *DDAH2*: dimethylarginine dimethylaminohydrolase 2

Gene		Case (STEMI) allele (n=296)		Control (Healthy) allele (n=150)		Significance		OR (95% CI)
		No.	%	No.	%	chi sq	p-value	
*NOS3* SNP (rs1799983)	G	179	60.5%	114	76.0%	10.62	0.001	0.48 (0.31-0.75)
	T	117	39.5%	36	24.0%	10.62	0.001	2.07 (1.33-3.22)
*DDAH2* SNP (rs805305)	G	168	56.8%	69	46.0%	4.63	0.032	1.54 (1.04-2.29)
	C	128	43.2%	81	54.0%	4.63	0.032	0.65 (0.44-0.96)

We analyzed the distribution of *NOS3* and *DDAH2* genetic variants across three groups: Group I (STEMI without diabetes, n=71), Group II (STEMI with diabetes, n=77), and Group III (healthy controls, n=75) (Table 4). For the *NOS3* SNP, the GG genotype was significantly less frequent in Groups I (43.7%) (n=31) and II (39.0%) (n=30) compared to controls (58.7%) (n=44) (p=0.020). The TT genotype was most prevalent in Group II (24.7%) (n=19) compared to Group I (15.5%) (n=11) and controls (6.7%) (n=5). The T allele frequency was significantly higher in Group II (42.9%) (n=66) than in Groups I (35.9%) (n=51) and III (24.0%) (n=36) (p=0.002).

**Table 4:** Comparison of NOS3 and DDAH2 genotypic variants among STEMI, STEMI with diabetes, and healthy controls Table showing genotypic (GG, GT, TT) frequencies of the *NOS3*-G894T polymorphism among STEMI (n=71), STEMI with diabetes (n=77), and healthy controls (n=75). Percentage comparison of genotypes (GG, GC, CC) of the *DDAH2* G/C polymorphism across the STEMI (n=71), STEMI with diabetes (n=77), and healthy controls (n=75). SNP: single nucleotide polymorphism; STEMI: ST-elevation myocardial infarction; DM: diabetes mellitus; *NOS3*: nitric oxide synthase; *DDAH2*: dimethylarginine dimethylaminohydrolase 2

Gene		Group I (STEMI) (n=71)		Group II (STEMI with DM) (n=77)		Group III (Healthy) (n=75)		Significance	
		No.	%	No.	%	No.	%	chi sq	p-value
*NOS3* SNP (rs1799983)	Genotype								
	Wild GG	31	43.7%	30	39.0%	44	58.7%	11.64	0.020
	Heterozygous mutant GT	29	40.8%	28	36.4%	26	34.7%		
	Homozygous mutant TT	11	15.5%	19	24.7%	5	6.7%		
*DDAH2* SNP (rs805305)	Genotype								
	Wild GG	21	29.6%	24	31.2%	15	20.0%	5.89	0.208
	Heterozygous mutant GC	40	56.3%	38	49.4%	39	52.0%		
	Homozygous mutant CC	10	14.1%	15	19.5%	21	28.0%		

For the *DDAH2* SNP, the GG genotype was more common in Groups I (29.6%) (n=21) and II (31.2%) (n=24) compared to controls (20.0%) (n=15). The CC genotype was least frequent in Group I (14.1%) (n=10) and most frequent in controls (28.0%) (n=21), though this difference was not significant (p=0.208). The G allele was more prevalent in Groups I (57.7%) (n=82) and II (55.8%) (n=86) compared to controls (46.0%) (n=69), with no significant association (p=0.094) (Table 5).

**Table 5:** Distribution of genotype allele frequency in NOS3 and DDAH2 between STEMI without diabetes, STEMI with diabetes, and healthy controls Table showing allele (G, T) frequencies of the *NOS3*-G894T polymorphism among STEMI (n=71), STEMI with diabetes (n=77), and healthy controls (n=75). Percentage comparison of allele (G, C) frequencies of the *DDAH2* G/C polymorphism across the STEMI (n=71), STEMI with diabetes (n=77), and healthy controls (n=75). SNP: single nucleotide polymorphism; STEMI: ST-elevation myocardial infarction; DM: diabetes mellitus*​​​​​​​; NOS3*: nitric oxide synthase; *DDAH2*: dimethylarginine dimethylaminohydrolase 2

Gene		Group I (STEMI) allele (n=142)		Group II (STEMI with DM) allele (n=154)		Group III (Healthy) allele (n=150)		Significance	
		No.	%	No.	%	No.	%	chi-sq	p-value
*NOS3* SNP (rs1799983)	Allele								
	G	91	64.1%	88	57.1%	114	76.0%	12.23	0.002
	T	51	35.9%	66	42.9%	36	24.0%		
*DDAH2* SNP (rs805305)	Allele								
	G	82	57.7%	86	55.8%	69	46.0%	4.73	0.094
	C	60	42.3%	68	44.2%	81	54.0%		

## Discussion

Genetic and environmental risk factors for CAD and STEMI have been widely studied [18,19]. CAD is a multifactorial and polygenic disease, but the role of *NOS3* and *DDAH2* polymorphisms in cardiac patients of Indian origin remains unclear. Notably, studies have presented that people of the Indian subcontinent have a higher susceptibility to CAD, where an increased incidence of the premature onset of CAD has been observed in Indians, and disease pathology occurs earlier than in different ethnic groups [20]. This study establishes an association between *NOS3* (rs1799983) and *DDAH2 *(rs805305) gene polymorphisms in STEMI patients from North India, highlighting their high prevalence in this population.

The endothelial isoform of nitric oxide synthase makes nitric oxide (NO) in endothelial cells. NO then goes into vascular smooth muscle cells and relaxes them. This helps keep the tone and structure of the blood vessels in balance. Because of this, eNOS is necessary for vascular homeostasis, and low NO levels caused by *NOS3* gene dysfunction lead to endothelial dysfunction, which is a key factor in STEMI development [21,22]. Mainly, L-arginine conversion produces endogenous NO, and the reaction is catalyzed by eNOS. NO is a highly lipophilic, diffusible, and gaseous signaling molecule that diffuses rapidly inside the target cells, mainly the vascular smooth muscle cells and neurons. Animal studies confirm that eNOS deficiency depletes NO production, underscoring its critical role in cardiovascular health [23,24]. In this study, the homozygous mutated TT genotype of the *NOS3* SNP was significantly more frequent in STEMI cases (20.3%) (n=30) than in healthy controls (6.7%) (n=5), strongly associating it with STEMI risk. On assessing the alleles, the G allele was less common in cases than in controls, while the T allele was significantly higher in cases when compared to controls, which presents a significant association. The higher prevalence of the T allele in cases further supports its role in CAD pathogenesis. Previous studies have demonstrated that the pathogenesis of STEMI can be due to various risk factors such as intake of sodium, alcohol consumption, obesity, and smoking. In addition, this study signifies that SNP findings of these genes in the given population can also be suggestive of CAD incidence.

The endogenous molecule asymmetric dimethylarginine (ADMA), an inhibitor of eNOS enzyme activity, leads to a decrease in NO concentration, which shows its significance in the pathogenesis of CAD. *DDAH2* enzyme hydrolyzes ADMA, promoting NO production and maintaining vascular health [25]. Studies in DDAH-null mice show that reduced DDAH expression increases ADMA levels, decreases NO, and raises blood pressure, suggesting that impaired *DDAH2 *activity may increase CAD risk [26,27].

This study found the GG genotype of the *DDAH2* SNP more prevalent in STEMI patients, while the CC genotype was less frequent in cases (16.9%) (n=25) than in controls (28.0%) (n=21), with a significant association that suggests that this homozygous mutated type may have a protective role in preventing CAD pathogenesis. Regarding the alleles, the G allele was more common in cases than in controls, while the C allele was significantly less frequent in cases compared to controls, with a significant association. These findings further link *DDAH2* polymorphisms to CAD. Results on *DDAH2 *polymorphisms have been mixed, with some studies showing associations with ADMA levels and CAD, while others, such as Maas et al., found no link [28], possibly due to differences in sample size, ethnicity, or regional genetic variations.

These findings suggest that different genotypic variants of *NOS3* and *DDAH2* genes can be used as a predictive marker and help identify STEMI patients, but further research is needed to refine diagnostic methods.

Limitations

This study provides valuable insights but has a few limitations. First, it primarily focuses on a specific ethnicity, limiting the generalizability of the findings across the globe. Broader studies with diverse and larger sample sizes are needed to address this limitation. Technically, while DNA sequencing remains the gold standard for identifying SNPs, its high cost poses a significant challenge in countries like India. Further research involving larger patient cohorts is essential to better define populations at risk for the *NOS3* and *DDAH2* SNPs. Using PCR-RFLP as a preliminary method to assess CAD risk at the genetic level, followed by sequencing, could provide a more comprehensive understanding of the disease’s underlying mechanisms.

## Conclusions

Identifying genetic markers such as *NOS3* and *DDAH2* polymorphisms can enhance early diagnosis and personalized treatment for STEMI patients. The study found a strong link between the G894T *NOS3* TT genotype and increased STEMI risk, especially in patients with diabetes. It also revealed an association between the *DDAH2* GG genotype and CAD, with the CC genotype offering a protective effect. These markers can aid in patient classification, improving prevention and treatment strategies.

## References

[1] Daiber A, Xia N, Steven S (2019). New therapeutic implications of endothelial nitric oxide synthase (eNOS) function/dysfunction in cardiovascular disease. Int J Mol Sci.

[2] Rizvi SMS, Mahdi F, Mahdi AA, Jafar T, Rizvi S (2020). Personalized medicine: role of asymmetric dimethylarginine as a predictive marker of CAD. Era's Journal of Medical Research.

[3] Torres M, Rosselló CA, Fernández-García P, Lladó V, Kakhlon O, Escribá PV (2020). The implications for cells of the lipid switches driven by protein-membrane interactions and the development of membrane lipid therapy. Int J Mol Sci.

[4] Tousoulis D, Kampoli AM, Tentolouris C, Papageorgiou N, Stefanadis C (2012). The role of nitric oxide on endothelial function. Curr Vasc Pharmacol.

[5] Zhu B, Si X, Gong Y (2017). An association between the endothelial nitric oxide synthase gene G894T polymorphism and premature coronary artery disease: a meta-analysis. Oncotarget.

[6] Nassereddine S, Hassani Idrissi H, Habbal R (2018). The polymorphism G894 T of endothelial nitric oxide synthase (eNOS) gene is associated with susceptibility to essential hypertension (EH) in Morocco. BMC Med Genet.

[7] Weiss SL, Yu M, Jennings L, Haymond S, Zhang G, Wainwright MS (2012). Pilot study of the association of the DDAH2 -449G polymorphism with asymmetric dimethylarginine and hemodynamic shock in pediatric sepsis. PLoS One.

[8] Abhary S, Burdon KP, Kuot A (2010). Sequence variation in DDAH1 and DDAH2 genes is strongly and additively associated with serum ADMA concentrations in individuals with type 2 diabetes. PLoS One.

[9] Corradi F, Bucciarelli B, Bianco F, Bucciarelli T (2024). Asymmetric dimethylarginine (ADMA) in cardiovascular disease, cardiac ischemia/reperfusion injury, and ischemic non-obstructive coronary artery disease: biochemical and pharmacological implications. Letters in Drug Design & Discovery.

[10] Assimes TL, Roberts R (2016). Genetics: Implications for prevention and management of coronary artery disease. J Am Coll Cardiol.

[11] Ray A, Maharana K, Meenakshi S, Singh S (2023). Endothelial dysfunction and its relation in different disorders: Recent update. Health Sciences Review.

[12] Oliveira-Paula GH, Lacchini R, Tanus-Santos JE (2016). Endothelial nitric oxide synthase: from biochemistry and gene structure to clinical implications of NOS3 polymorphisms. Gene.

[13] Król M, Kepinska M (2020). Human nitric oxide synthase—Its functions, polymorphisms, and inhibitors in the context of inflammation, diabetes and cardiovascular diseases. Int J Mol Sci.

[14] Caplin B, Leiper J (2012). Endogenous nitric oxide synthase inhibitors in the biology of disease: markers, mediators, and regulators?. Arterioscler Thromb Vasc Biol.

[15] Shiraz Rizvi SM, Sunny S, Wani IA, Mahdi F, Zaidi ZH, Rajasekaran NS (2023). Influence of electrolyte imbalance on regional wall motion abnormalities in STEMI patients of North Indian origin. Front Cardiovasc Med.

[16] Smith SW, Dodd KW, Henry TD, Dvorak DM, Pearce LA (2012). Diagnosis of ST-elevation myocardial infarction in the presence of left bundle branch block with the ST-elevation to S-wave ratio in a modified Sgarbossa rule. Ann Emerg Med.

[17] Xu AG, Xu RM, Lu CQ (2012). Association study of dimethylarginine dimethylaminohydrolase 2 gene polymorphisms and coronary heart disease. Mol Med Rep.

[18] Žaliaduonytė-Pekšienė D, Lesauskaitė V, Liutkevičienė R (2017). Association of the genetic and traditional risk factors of ischaemic heart disease with STEMI and NSTEMI development. J Renin Angiotensin Aldosterone Syst.

[19] Dai X, Wiernek S, Evans JP, Runge MS (2016). Genetics of coronary artery disease and myocardial infarction. World J Cardiol.

[20] Trimèche-Othmani T, Kammoun M, Callebert J (2016). Association between genetic polymorphisms of ACE, eNOS, DDAH-2 and ADMA, SDMA and NOx with coronary artery disease in Tunisian population. Int J Clin Exp Med.

[21] Severino P, D'Amato A, Prosperi S (2021). Potential role of eNOS genetic variants in ischemic heart disease susceptibility and clinical presentation. J Cardiovasc Dev Dis.

[22] Janssens S, Pokreisz P, Schoonjans L (2004). Cardiomyocyte-specific overexpression of nitric oxide synthase 3 improves left ventricular performance and reduces compensatory hypertrophy after myocardial infarction. Circ Res.

[23] Liu VW, Huang PL (2008). Cardiovascular roles of nitric oxide: a review of insights from nitric oxide synthase gene disrupted mice. Cardiovasc Res.

[24] Kim GH, Ryan JJ, Archer SL (2013). The role of redox signaling in epigenetics and cardiovascular disease. Antioxid Redox Signal.

[25] Gawrys J, Gajecki D, Szahidewicz-Krupska E, Doroszko A (2020). Intraplatelet L-arginine-nitric oxide metabolic pathway: from discovery to clinical implications in prevention and treatment of cardiovascular disorders. Oxid Med Cell Longev.

[26] Lai L, Ghebremariam YT (2016). Modulating DDAH/NOS pathway to discover vasoprotective insulin sensitizers. J Diabetes Res.

[27] Quintana-Villamandos B, Arnalich-Montiel A, Arribas S (2016). Early regression of coronary artery remodeling with esmolol and DDAH/ADMA pathway in hypertensive rats. Hypertens Res.

[28] Maas R, Erdmann J, Lüneburg N (2009). Polymorphisms in the promoter region of the dimethylarginine dimethylaminohydrolase 2 gene are associated with prevalence of hypertension. Pharmacol Res.

